# 5-Chloro­indoline-2,3-dione

**DOI:** 10.1107/S1600536810042522

**Published:** 2010-10-31

**Authors:** Wen-Bin Wei, Shuo Tian, Hao Zhou, Jie Sun, Hai-Bo Wang

**Affiliations:** a College of Light Industry and Food Science, Nanjing University of Technology, Xinmofan Road No. 5 Nanjing, Nanjing 210009, People’s Republic of China; b College of Science, Nanjing University of Technology, Xinmofan Road No. 5 Nanjing, Nanjing 210009, People’s Republic of China

## Abstract

The title compound, C_8_H_4_ClNO_2_, is almost planar (r.m.s. deviation for the non-H atoms = 0.023 Å). In the crystal, N—H⋯O hydrogen bonds link the mol­ecules into *C*(4) chains propagating in [001] and C—H⋯O inter­actions cross-link the chains.

## Related literature

For further synthetic details, see: Silva *et al.* (2001 ▶). For reference bond lengths, see: Allen *et al.* (1987 ▶).
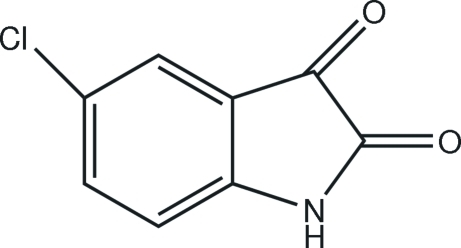


## Experimental

### 

#### Crystal data

C_8_H_4_ClNO_2_
*M* *_r_* = 181.57
Orthorhombic, 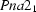

*a* = 24.706 (5) Å
*b* = 5.6890 (11) Å
*c* = 5.209 (1) Å
*V* = 732.1 (2) Å^3^
*Z* = 4
Mo *K*α radiation
μ = 0.47 mm^−1^
*T* = 293 K
0.10 × 0.05 × 0.05 mm

#### Data collection

Enraf–Nonius CAD-4 diffractometer
Absorption correction: ψ scan (North *et al.*, 1968 ▶) *T*_min_ = 0.955, *T*_max_ = 0.977
1746 measured reflections
884 independent reflections
734 reflections with *I* > 2σ(*I*)
*R*_int_ = 0.048
3 standard reflections every 200 reflections  intensity decay: 1%

#### Refinement

*R*[*F* ^2^ > 2σ(*F* ^2^)] = 0.037
*wR*(*F* ^2^) = 0.102
*S* = 1.00
884 reflections
109 parameters
2 restraints
H-atom parameters constrained
Δρ_max_ = 0.18 e Å^−3^
Δρ_min_ = −0.24 e Å^−3^
Absolute structure: Flack (1983 ▶), 862 Friedel pairs
Flack parameter: 0.11 (16)

### 

Data collection: *CAD-4 EXPRESS* (Enraf–Nonius, 1994 ▶); cell refinement: *CAD-4 EXPRESS*; data reduction: *XCAD4* (Harms & Wocadlo, 1995 ▶); program(s) used to solve structure: *SHELXS97* (Sheldrick, 2008 ▶); program(s) used to refine structure: *SHELXL97* (Sheldrick, 2008 ▶); molecular graphics: *SHELXTL* (Sheldrick, 2008 ▶); software used to prepare material for publication: *SHELXL97*.

## Supplementary Material

Crystal structure: contains datablocks global, I. DOI: 10.1107/S1600536810042522/hb5688sup1.cif

Structure factors: contains datablocks I. DOI: 10.1107/S1600536810042522/hb5688Isup2.hkl

Additional supplementary materials:  crystallographic information; 3D view; checkCIF report

## Figures and Tables

**Table 1:** Hydrogen-bond geometry (Å, °)

*D*—H⋯*A*	*D*—H	H⋯*A*	*D*⋯*A*	*D*—H⋯*A*
N—H0*A*⋯O1^i^	0.86	2.04	2.893 (4)	172
C7—H7*A*⋯O2^ii^	0.93	2.39	3.301 (5)	166

Symmetry codes: (i) 
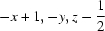
; (ii) 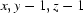.

**Fig. 1.**
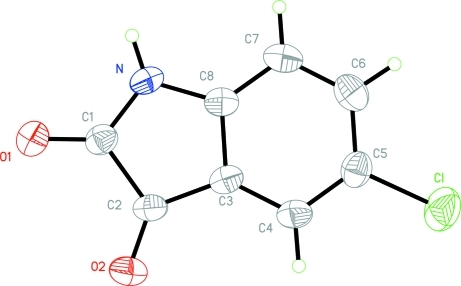
View of the title compound with displacement ellipsoids for non-H atoms drawn at the 50% probability level.

**Fig. 2.**
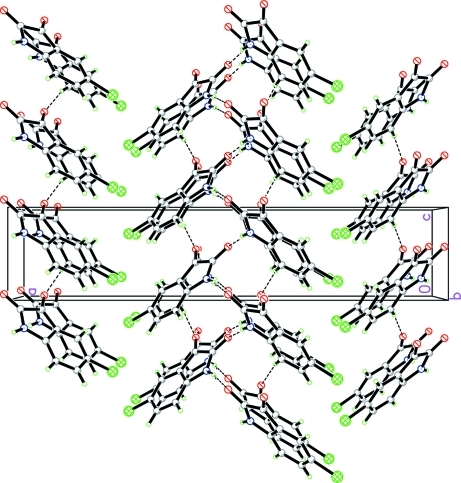
Packing diagram.

## supplementary crystallographic information

### Comment

5-Chloroindoline-2,3-dione is an important pharmaceutical intermediate for synthesizing 5-chlorooxindole and tenidap which was evaluated as novel nonsteroidal anti-inflammatory agents. We report herein the crystal structure of the title compound.

In the molecule of the title compound (Fig 1), the bond lengths (Allen *et al.*, 1987) and angles are within normal ranges. Rings A (N/C1—C3/C8) and B (C3—C8) are nearly coplanar, and they are oriented at dihedral angles of A/B = 0.30 (3).

In the crystal structure, intermolecular N—H···O interaction may be effective in the stabilization of the structure.

### Experimental

For the preparation of the title compound, the method developed by Sandmeyer is the oldest and the most frequently used. It consists in the reaction of 4-chloroaniline with chloral hydrate and hydroxylamine hydrochloride in aqueous sodium sulfate to form an 4-chloroisonitrosoacetanilide, which after isolation, when treated with concentrated sulfuric acid, furnishes the title compound in 75% overall yield (Silva *et al.*, 2001). Red blocks of (I) were obtained by slow evaporation of a methanol solution (m.p. 520 K).

### Refinement

H atoms were positioned geometrically, with N—H = 0.86 Å (for NH) and C—H = 0.93 Å for aromatic, respectively, and constrained to ride on their parent atoms, with *U*_iso_(H) = *xU*_eq_(C,*N*), where *x* = 1.5 for NH H and *x* = 1.2 for all other H atoms.

### Crystal data

**Table:**

C_8_H_4_ClNO_2_	*D*_x_ = 1.647 Mg m^−^^3^
*M**_r_* = 181.57	Melting point: 520 K
Orthorhombic, *P**n**a*2_1_	Mo *K*α radiation, λ = 0.71073 Å
Hall symbol: P 2c -2n	Cell parameters from 25 reflections
*a* = 24.706 (5) Å	θ = 9–13°
*b* = 5.6890 (11) Å	µ = 0.47 mm^−^^1^
*c* = 5.209 (1) Å	*T* = 293 K
*V* = 732.1 (2) Å^3^	Block, red
*Z* = 4	0.10 × 0.05 × 0.05 mm
*F*(000) = 368	

### Data collection

**Table:**

Enraf–Nonius CAD-4 diffractometer	734 reflections with *I* > 2σ(*I*)
Radiation source: fine-focus sealed tube	*R*_int_ = 0.048
graphite	θ_max_ = 27.0°, θ_min_ = 1.7°
ω/2θ scans	*h* = −31→31
Absorption correction: ψ scan (North *et al.*, 1968)	*k* = −7→0
*T*_min_ = 0.955, *T*_max_ = 0.977	*l* = 0→6
1746 measured reflections	3 standard reflections every 200 reflections
884 independent reflections	intensity decay: 1%

### Refinement

**Table:**

Refinement on *F*^2^	Secondary atom site location: difference Fourier map
Least-squares matrix: full	Hydrogen site location: inferred from neighbouring sites
*R*[*F*^2^ > 2σ(*F*^2^)] = 0.037	H-atom parameters constrained
*wR*(*F*^2^) = 0.102	*w* = 1/[σ^2^(*F*_o_^2^) + (0.065*P*)^2^] where *P* = (*F*_o_^2^ + 2*F*_c_^2^)/3
*S* = 1.00	(Δ/σ)_max_ < 0.001
884 reflections	Δρ_max_ = 0.18 e Å^−^^3^
109 parameters	Δρ_min_ = −0.24 e Å^−^^3^
2 restraints	Absolute structure: Flack (1983), 862 Friedel pairs
Primary atom site location: structure-invariant direct methods	Flack parameter: 0.11 (16)

### Special details

**Table:**

Geometry. All e.s.d.'s (except the e.s.d. in the dihedral angle between two l.s. planes) are estimated using the full covariance matrix. The cell e.s.d.'s are taken into account individually in the estimation of e.s.d.'s in distances, angles and torsion angles; correlations between e.s.d.'s in cell parameters are only used when they are defined by crystal symmetry. An approximate (isotropic) treatment of cell e.s.d.'s is used for estimating e.s.d.'s involving l.s. planes.
Refinement. Refinement of *F*^2^ against ALL reflections. The weighted *R*-factor *wR* and goodness of fit *S* are based on *F*^2^, conventional *R*-factors *R* are based on *F*, with *F* set to zero for negative *F*^2^. The threshold expression of *F*^2^ > σ(*F*^2^) is used only for calculating *R*-factors(gt) *etc*. and is not relevant to the choice of reflections for refinement. *R*-factors based on *F*^2^ are statistically about twice as large as those based on *F*, and *R*- factors based on ALL data will be even larger.

### Fractional atomic coordinates and isotropic or equivalent isotropic displacement parameters (Å^2^)

**Table:**

	*x*	*y*	*z*	*U*_iso_*/*U*_eq_	
Cl	0.26410 (4)	0.6028 (2)	0.2215 (3)	0.0588 (4)	
N	0.45038 (11)	0.1571 (5)	0.7251 (9)	0.0378 (8)	
H0A	0.4676	0.0291	0.6924	0.045*	
O1	0.49964 (12)	0.2818 (5)	1.0752 (7)	0.0441 (7)	
C1	0.46351 (13)	0.3075 (6)	0.9153 (9)	0.0335 (8)	
O2	0.42293 (11)	0.6781 (5)	1.0449 (7)	0.0446 (7)	
C2	0.42335 (13)	0.5098 (6)	0.9018 (8)	0.0332 (8)	
C3	0.38713 (13)	0.4525 (6)	0.6867 (8)	0.0321 (8)	
C4	0.34278 (14)	0.5650 (7)	0.5820 (9)	0.0351 (9)	
H4A	0.3300	0.7058	0.6497	0.042*	
C5	0.31808 (13)	0.4609 (7)	0.3733 (8)	0.0373 (9)	
C6	0.33568 (15)	0.2468 (7)	0.2760 (8)	0.0409 (10)	
H6A	0.3175	0.1793	0.1381	0.049*	
C7	0.37976 (16)	0.1329 (6)	0.3811 (10)	0.0390 (9)	
H7A	0.3920	−0.0093	0.3148	0.047*	
C8	0.40480 (13)	0.2366 (6)	0.5864 (9)	0.0333 (8)	

### Atomic displacement parameters (Å^2^)

**Table:**

	*U*^11^	*U*^22^	*U*^33^	*U*^12^	*U*^13^	*U*^23^
Cl	0.0493 (6)	0.0757 (8)	0.0512 (6)	0.0147 (5)	−0.0117 (6)	0.0048 (8)
N	0.0425 (16)	0.0279 (14)	0.0430 (19)	0.0100 (12)	−0.0008 (19)	−0.002 (2)
O1	0.0477 (13)	0.0415 (14)	0.0431 (17)	0.0070 (13)	−0.0070 (15)	0.0018 (16)
C1	0.0351 (18)	0.0309 (18)	0.034 (2)	0.0025 (15)	0.0050 (19)	0.0035 (19)
O2	0.0524 (16)	0.0387 (14)	0.0427 (17)	0.0069 (13)	0.0030 (15)	−0.0108 (15)
C2	0.0398 (18)	0.0263 (15)	0.033 (2)	0.0039 (15)	0.0081 (18)	−0.0007 (18)
C3	0.0341 (16)	0.0284 (15)	0.034 (2)	0.0043 (14)	0.0069 (18)	0.0027 (17)
C4	0.0402 (18)	0.0330 (17)	0.032 (2)	0.0046 (15)	0.0074 (18)	−0.0012 (18)
C5	0.0326 (16)	0.045 (2)	0.034 (2)	0.0015 (16)	0.0002 (18)	0.006 (2)
C6	0.0439 (19)	0.045 (2)	0.033 (2)	−0.0096 (18)	0.0013 (18)	−0.0013 (19)
C7	0.049 (2)	0.0302 (17)	0.038 (2)	−0.0020 (16)	0.009 (2)	−0.0053 (18)
C8	0.0352 (17)	0.0294 (17)	0.035 (2)	−0.0004 (15)	0.0059 (18)	−0.0018 (18)

### Geometric parameters (Å, °)

**Table:**

Cl—C5	1.748 (4)	C3—C8	1.404 (5)
N—C1	1.349 (6)	C4—C5	1.381 (6)
N—C8	1.412 (5)	C4—H4A	0.9300
N—H0A	0.8600	C5—C6	1.389 (6)
O1—C1	1.229 (5)	C6—C7	1.380 (6)
C1—C2	1.521 (4)	C6—H6A	0.9300
O2—C2	1.213 (4)	C7—C8	1.369 (6)
C2—C3	1.471 (5)	C7—H7A	0.9300
C3—C4	1.381 (5)		
			
C1—N—C8	111.4 (3)	C3—C4—H4A	121.2
C1—N—H0A	124.3	C4—C5—C6	121.7 (4)
C8—N—H0A	124.3	C4—C5—Cl	119.7 (3)
O1—C1—N	126.7 (3)	C6—C5—Cl	118.6 (3)
O1—C1—C2	126.5 (4)	C7—C6—C5	120.9 (4)
N—C1—C2	106.8 (3)	C7—C6—H6A	119.5
O2—C2—C3	129.6 (3)	C5—C6—H6A	119.5
O2—C2—C1	125.1 (4)	C8—C7—C6	117.6 (4)
C3—C2—C1	105.3 (3)	C8—C7—H7A	121.2
C4—C3—C8	120.3 (4)	C6—C7—H7A	121.2
C4—C3—C2	132.9 (3)	C7—C8—C3	121.8 (4)
C8—C3—C2	106.7 (3)	C7—C8—N	128.5 (3)
C5—C4—C3	117.6 (4)	C3—C8—N	109.7 (4)
C5—C4—H4A	121.2		
			
C8—N—C1—O1	176.7 (4)	C3—C4—C5—Cl	−176.5 (3)
C8—N—C1—C2	−0.8 (4)	C4—C5—C6—C7	−1.8 (6)
O1—C1—C2—O2	2.4 (7)	Cl—C5—C6—C7	176.9 (3)
N—C1—C2—O2	179.9 (4)	C5—C6—C7—C8	1.0 (6)
O1—C1—C2—C3	−177.1 (4)	C6—C7—C8—C3	−0.7 (6)
N—C1—C2—C3	0.4 (4)	C6—C7—C8—N	−179.5 (4)
O2—C2—C3—C4	−0.2 (7)	C4—C3—C8—C7	1.2 (6)
C1—C2—C3—C4	179.2 (4)	C2—C3—C8—C7	−179.6 (4)
O2—C2—C3—C8	−179.3 (4)	C4—C3—C8—N	−179.8 (4)
C1—C2—C3—C8	0.2 (4)	C2—C3—C8—N	−0.6 (4)
C8—C3—C4—C5	−1.9 (6)	C1—N—C8—C7	179.8 (4)
C2—C3—C4—C5	179.2 (4)	C1—N—C8—C3	0.9 (5)
C3—C4—C5—C6	2.2 (6)		

### Hydrogen-bond geometry (Å, °)

**Table:**

*D*—H···*A*	*D*—H	H···*A*	*D*···*A*	*D*—H···*A*
N—H0A···O1^i^	0.86	2.04	2.893 (4)	172
C7—H7A···O2^ii^	0.93	2.39	3.301 (5)	166

Symmetry codes: (i) −*x*+1, −*y*, *z*−1/2; (ii) *x*, *y*−1, *z*−1.
